# Severity of Depressive Symptoms and Accuracy of Dietary Reporting among Obese Women with Major Depressive Disorder Seeking Weight Loss Treatment

**DOI:** 10.1371/journal.pone.0090361

**Published:** 2014-02-28

**Authors:** Matthew C. Whited, Kristin L. Schneider, Bradley M. Appelhans, Yunsheng Ma, Molly E. Waring, Michele A. DeBiasse, Andrew M. Busch, Jessica L. Oleski, Philip A. Merriam, Barbara C. Olendzki, Sybil L. Crawford, Ira S. Ockene, Stephenie C. Lemon, Sherry L. Pagoto

**Affiliations:** 1 Department of Psychology, East Carolina University, Greenville, North Carolina, United States of America; 2 Department of Psychology, Rosalind Franklin University, Chicago, Illinois, United States of America; 3 Departments of Preventive Medicine and Behavioral Sciences, Rush University Medical Center, Chicago, Illinois, United States of America; 4 Department of Medicine, Division of Preventive and Behavioral Medicine, University of Massachusetts Medical School, Worcester, Massachusetts, United States of America,; 5 Division of Epidemiology of Chronic Diseases and Vulnerable Populations, Department of Quantitative Health Sciences, University of Massachusetts Medical School, Worcester, Massachusetts, United States of America; 6 Boston University, College of Health & Rehabilitation Sciences, Sargent Department of Health Sciences Boston, Massachusetts, United States of America; 7 Centers for Behavioral and Preventive Medicine, The Miriam Hospital and the Alpert Medical School of Brown University, Providence, Rhode Island, United States of America; 8 Department of Medicine, Division of Cardiovascular Medicine, University of Massachusetts,Medical School, Worcester, Massachusetts, United States of America; Federal University of Rio de Janeiro, Brazil

## Abstract

An elevation in symptoms of depression has previously been associated with greater accuracy of reported dietary intake, however this association has not been investigated among individuals with a diagnosis of major depressive disorder. The purpose of this study was to investigate reporting accuracy of dietary intake among a group of women with major depressive disorder in order to determine if reporting accuracy is similarly associated with depressive symptoms among depressed women. Reporting accuracy of dietary intake was calculated based on three 24-hour phone-delivered dietary recalls from the baseline phase of a randomized trial of weight loss treatment for 161 obese women with major depressive disorder. Regression models indicated that higher severity of depressive symptoms was associated with greater reporting accuracy, even when controlling for other factors traditionally associated with reporting accuracy (coefficient  =  0.01 95% CI = 0.01 – 0.02). Seventeen percent of the sample was classified as low energy reporters. Reporting accuracy of dietary intake increases along with depressive symptoms, even among individuals with major depressive disorder. These results suggest that any study investigating associations between diet quality and depression should also include an index of reporting accuracy of dietary intake as accuracy varies with the severity of depressive symptoms.

## Introduction

Weight loss, especially in the context of individuals who are experiencing depressive symptoms, can be challenging. Individuals with significant depressive symptoms lose less weight than what is typically observed among those who do not, though individuals with significant depressive symptoms do show improvements in their weight in response to weight loss treatment [1], [2]. A key feature of weight management programs is the self-monitoring of dietary intake [3], which provides clinicians with the information they need to guide a patient in making healthier choices and losing weight. Studies using doubly-labeled water to estimate energy expenditure suggest that non-depressed individuals underestimate their daily energy intake by 10-40% [4], [5]. This is concerning from the standpoint of clinical care and research that aims to understand energy balance. Assessing reporting accuracy for dietary intake could inform research and subsequent practice and policy recommendations, especially if reporting accuracy varies across different subsets of a given population. Characteristics previously associated with low reporting accuracy for dietary intake include higher body mass index (BMI) [6]-[8], lower education, lower income, dietary restriction, and smoking status [6], and inconsistently, female gender [6], [9]. More accurate reporting of dietary intake has also been associated with higher report of depression symptoms [7], [10].

Major depressive disorder is a psychiatric condition characterized by persistent sadness, or loss of interest in enjoyable activities, and other symptoms such as appetite disturbance, sleep disturbance, fatigue, difficulty concentrating, feelings of hopelessness, or agitation that significantly interfere with functioning [11]. Depression is more prevalent among women than men [12], [13], and is highly comorbid with obesity [14]-[16]. Some research asserts that individuals with major depressive disorder have a less healthy diet [17]. Prior studies have observed greater reporting accuracy among women with higher depressive symptoms [7], [10], but looked at this association in samples not selected for depression or depressive symptoms [7], [9], [10]. For example, in a study by Kretsch et al [7], who used an early version of the Beck Depression Inventory, the mean score was 3.5 (SD = 2.1) out of 63 among obese women. This score is indicative of a minimal level of depressive symptoms. Meeting diagnostic criteria for major depressive disorder not only involves more severe levels of depressive symptoms than what is observed in prior studies of depressive symptoms and dietary reporting accuracy [7], [9], [10], but a diagnosis also requires evidence that these symptoms cause significant distress and/or interfere with social, occupational, or other important areas of functioning [11]. Individuals with this level of impairment may also be likely to experience difficulty reporting their dietary intake, especially due to symptoms of fatigue and difficulty concentrating. The association previously observed between higher reporting accuracy and higher depressive symptom severity may not be observed among individuals with a diagnosis of major depressive disorder.

Reporting accuracy among individuals with a major depressive disorder diagnosis also impacts research on mood and diet quality, as major depressive disorder [17] and severity of depressive symptoms [18] have been associated with lower diet quality. The importance of considering dietary reporting accuracy in any study investigating dietary intake was highlighted in a recent article that demonstrated increased reporting accuracy was the reason for the observed increase in energy intake over 39 years of the National Health and Nutrition Examination Survey [8]. The authors of this study point out that ignorance of the impact of dietary reporting accuracy on reported energy intake has resulted in, perhaps incorrectly, attributing the obesity epidemic to increased average energy intake by the US population. The same reasoning could apply to depression and dietary intake, such that poorer diet or higher energy intake could be attributed to the presence of depression or more severe depressive symptoms and neglect the influence of depressive symptoms on reporting accuracy. A greater understanding of the relationship between depressive symptom severity and reporting accuracy is needed to inform this research.

No prior studies have investigated reporting accuracy among individuals with a diagnosis of major depressive disorder. The previously observed association between higher depressive symptoms and greater reporting accuracy may continue in a clinical sample with higher levels of depressive symptoms than those observed in previous studies, but this is unclear due to the level of impairment these individuals experience due to their symptoms of depression. The aim of the current study was to examine the association between depression severity and dietary reporting accuracy in obese women with a diagnosis of major depressive disorder, and also examine the prevalence of low energy reporting [19] among this population.

## Methods

### Ethics Statement

All participants provided written informed consent, and all procedures were approved by the Institutional Review Board of the University of Massachusetts Medical School, which considered all medical and psychological ethical issues.

### Participants

Participants were 161 women who completed the baseline assessment for a randomized clinical trial (ClinicalTrials.gov: NCT00572520) investigating a behavioral intervention for co-morbid obesity and major depressive disorder [1]. The current study is a secondary analysis of the baseline phase of this clinical trial. The baseline phase of the trial was chosen in order to account for participants’ attempts to lose weight, to varying degrees of success, as part of the intervention phase of the trial. The design and methods of this trial, including all inclusion and exclusion criteria, have been described in detail [20]. Briefly, participants were recruited from primary care clinics and the local community between July 2007 and March 2010. Inclusion criteria were: age between 21 and 65 years; a current diagnosis of major depressive disorder via the Structured Clinical Interview for DSM-IV [21]; a Hamilton Rating Scale for Depression (HRSD [22]) score from 12-26; and a body mass index (BMI) of 30-40 kg/m^2^. Individuals with other psychological diagnoses that would require additional treatment beyond the study protocol (i.e. psychotic disorders, bipolar disorders, post-traumatic stress disorder, anorexia nervosa, bulimia nervosa) were excluded. Participants had to be considered healthy enough to engage in a standard weight loss treatment program that included diet modification and exercise, and permission to participate from their primary care providers was required.

### Procedure

Participants were screened via phone for self-reported depression status and BMI. Eligible participants were invited for an in-person screening during which they provided written informed consent, underwent a diagnostic clinical interview (SCID-IV [21]), had height and weight measurements taken, and completed demographics and medical history questionnaires. During the baseline phase of the study, participants completed three telephone 24-hour dietary and physical activity recalls. The phone calls were unscheduled and at least one occurred on a weekday and one on a weekend day. Participants were instructed to continue with their normal diet and exercise patterns during the baseline phase of the study.

### Measures

Anthropometric Measures included weight and height measured using a digital scale and stadiometer. BMI was calculated as weight (kg) divided by height squared (m^2^).

Diet and physical activity recalls assessed all food and beverages consumed over the past 24 hours. Three dietary recalls provide adequate assessment of energy intake when calls are spread across both weekend and weekdays [23]. Participants were phoned at unscheduled times within two weeks of their baseline session. A trained registered dietitian used a computer-guided multiple-pass technique to thoroughly assess dietary intake. Participants used a reference booklet that was provided at the screening session to aid food portion size estimations. The Nutrition Data System for Research (NDSR; version 2010, Nutrition Coordinating Center, University of Minnesota, MN) was used to determine dietary intake based on the foods, food amounts, and preparation used by the participants. From the information provided by each participant, average daily energy intake was calculated. Participants also recalled the time spent in mild, moderate, hard, and very hard physical activity across multiple domains: sports/exercise/leisure, household, and occupational activity. Average daily energy expenditure (METs) was calculated based on the time each participant spent in each activity.

Dietary reporting accuracy was calculated as ratio of reported energy intake to resting metabolic rate (EI_rep_:RMR). This ratio provides an index of accuracy, with a higher value representing more accurate reporting [24]-[26]. This ratio can be further refined by multiplying RMR by a physical activity coefficient to estimate total energy expenditure (TEE) resulting in an EI­_rep_:TEE value. We used a coefficient of 1.20, as suggested by previous research among sedentary populations [24], and chose the Mifflin-St.Jeor equation to calculate RMR as it is the most accurate among otherwise healthy obese individuals [26].

Participants were further categorized as low-energy-, over-, or accurate reporters using the Goldberg cut-offs and procedures described by Tooze and colleagues [19]. This procedure provides an acceptable estimation of reporting accuracy in the absence of doubly labeled water data [19]. According to this procedure we constructed the 95% confidence limit based on an average physical activity coefficient of 1.55 and 3 days of dietary assessment. Individuals with an EI_rep_:RMR ratio below the 95% confidence interval are considered low energy reporters and those above are considered over-reporters.

Depressive symptom severity at baseline was assessed using the Beck Depression Inventory II (BDI-II) [27]. The BDI-II is a 21-item self-report assessment of the severity of depressive symptoms. Each item is rated on a scale from 0-3 with a total score ranging from 0-63, with higher scores indicating more severe depressive symptoms.

To determine if depression severity is related to reporting accuracy among our sample of obese women with major depressive disorder seeking weight-loss treatment, EI_rep_:TEE value was used as a continuous dependent variable in multiple regression. Over-reporters were excluded (n = 5) so that EI­_rep_:TEE would accurately represent an increasing level of accuracy. Linear regression models were conducted to determine the unadjusted association between depression severity and reporting accuracy, and the multivariate association controlling for traditional factors related to reporting accuracy (i.e. education, income, BMI). Other potentially confounding factors were controlled by the constitution of the parent study, which only included women, all of whom reported currently being non-smokers. Cutoffs for low-energy reporting were used to provide descriptive data regarding the prevalence of low-energy reporting in the sample. A logistic regression model explored the association between depression severity and low-energy reporting, controlling for education, income, and BMI. Analyses were conducted using SPSS (Version 19, Armonk, NY: IBM Corp).

## Results

Sample characteristics are presented in Table 1. The mean BDI-II score was 21.1 (SD = 5.8) indicating a moderate level of depressive symptoms. The average energy expenditure of our sample was 27.90 MET hours/day (SD = 3.97), which indicates our sample was sedentary on average. Greater depression severity was related to greater reporting accuracy (coefficient  =  0.01; 95% CI: 0.003-0.02, Model 1, Table 2). This association did not change with adjustment for education, income, and BMI (Model 2, Table 2). The 95% confidence limit for estimating accurate reporting was 1.00 - 2.40; participants above or below this confidence limit were classified as low-energy or over-reporters, respectively. Seventeen percent (n = 27) of women were classified as low-energy reporters; 5 were classified as over-reporters. Depressive severity was associated with under-reporting of energy intake; for every one-point higher BDI score, women had 8% lower odds of being a low-energy reporter (OR  =  0.92; 95% CI: 0.85-1.0).

**Table 1:** Sample characteristics.

	Mean (SD) or percent
**N**	161
**Age (years)**	45.9 (10.8)
**BMI**	35.4 (3.3)
**Marital status**	
*married*	59.0%
*not married*	41.0%
**Annual household income (U.S. dollars)**	
*under $40,000*	21.7%
*$40,000-$75,000*	42.2%
*over 75,000*	36.0%
**Education**	
*< Bachelors degree*	54.0%
*Bachelors degree*	30.4%
*Postgraduate degree*	15.5%
**Hispanic/Latino ethnicity**	9.9%
**Race**	
*Caucasian*	85.1%
*African American*	4.3%
*Asian*	1.2%
*Native American/Alaskan Native*	6.2%
*Multi-Racial*	3.1%

**Table 2:** Linear regression analyses of the association between depression severity and reporting accuracy of dietary intake among women with comorbid obesity and major depressive disorder^a^.

	*Coefficient (95% CI)*
**Model 1**	
BDI-II†	**0.01 (0.003 – 0.02)**
**Model 2**	
*Education*	
< Bachelors degree	Reference
Bachelors degree	0.01 (-0.01 – 0.21)
Postgraduate degree	0.11 (-0.03 – 0.24)
*Annual household income*	
under $40,000	Reference
$40,000-$75,000	0.10 (-0.02 – 0.23)
over 75,000	0.02 (-0.11 – 0.14)
*BMI* ‡ *(kg/m^2^)*	-0.006 (-0.02 – 0.01)
*BDI-II* †	**0.01 (0.01 – 0.02)**

a Reporting accuracy expressed as the ratio of reported energy intake to total energy expenditure (EI_rep_:TEE). TEE calculated based on the participant’s weight, height, and age. Depression severity measured by the Beck Depression Inventory II.

† BDI-II, Beck Depression Inventory II.

‡ BMI, Body Mass Index.

Significant associations as indicated by 95% confidence intervals are highlighted in **bold**.

## Discussion

The current study found a positive association between depression severity and reporting accuracy of dietary intake among women with comorbid obesity and major depressive disorder. These results confirm those of other studies that have found associations between higher depressive symptoms and greater reporting accuracy in euthymic samples [7], [10] and expands this finding to a population of women with comorbid obesity and major depressive disorder. Individuals with major depressive disorder experience significant functional impairment as a result of depressive symptoms, and so increasing accuracy with increasing depressive symptoms is somewhat surprising. In addition, the prevalence of low energy reporting in our sample (17%) was low compared to the prevalence observed among non-depressed samples of patients with other chronic medical illnesses that used similar methods of assessing low energy reporting [24], [28], and in comparison to US population samples [8]. The observed findings have implications for future research on diet in the context of depression. Studies have observed poorer diet quality among participants with major depressive disorder as compared to non-depressed groups [17]. It is possible that individuals with more severe depression symptomatology appear to have poorer quality diets, and higher energy intake, in part because they are more accurate reporters of their dietary intake. However; reporting accuracy would need to be included in future studies of the relationship between depressive symptoms and diet quality in order to confirm this speculation.

Despite the results of previous studies, it is surprising that a positive association between depressive symptom severity emerged among a sample with more severe depressive symptoms, as increasing depressive symptoms are typically related to poorer performance across many other domains (e.g., cognitive functioning, concentration), which could impact reporting accuracy. One potential explanation for increased reporting accuracy along with increasing depressive symptoms is a lower level of social desirability. The association between depressive symptoms, especially hopelessness, and social desirability is negative, such that individuals who report higher depressive symptoms report lower need to appear socially desirable [29]-[31]. It is possible that individuals in our sample with higher depressive symptoms were less likely to underestimate their caloric intake in order to make a positive impression on the intervention leaders. Another possible explanation for better reporting accuracy among those with high depressive symptoms is the phenomenon known as depressive realism which states that depressed individuals have a more accurate perception of reality compared to non-depressed individuals, who tend to demonstrate an optimistic bias [32], [33]. The generalizability of his phenomenon has been debated across the psychological literature, but one consistent finding in laboratory studies has been that depressed participants tend to be more accurate in their reports of negative information compared to participants without depression [34]. If high caloric intake functioned as negative information for the participants in the current study, more severely depressed participants may have been less biased in their recall because they were less susceptible to an optimistic bias regarding their eating habits.

Reporting accuracy was assessed only prior to the start of weight loss treatment in this study; thus, suggesting alterations of weight loss treatments for women with comorbid obesity and depression based on the results of this study would be premature. Also, reported dietary intake was assessed via telephone phone using structured recall, which differs from the method of self-report typically utilized in a weight loss treatment setting. However; if future research among clinical samples confirms that obese women with depression are more accurate as they progress through treatment, then clinicians may adjust their feedback to these patients accordingly. For example, a counselor may be able suggest additional reductions in caloric intake based around specific foods using the information provided by more accurate dietary reporting. This may be an especially important strategy among patients with major depressive disorder due to difficulties with weight loss in this population. Due to the fact that the current study was a secondary analysis of a randomized controlled trial, the generalizability of our findings are limited by the exclusion criteria of the parent study; participants with more severe depression (HRSD scores>26), a BMI under 30 or over 40 kg/m^2^, and men were not included. A small sample size also limited this study, and no a priori power analysis was conducted specifically for this analysis. Our sample was predominantly Caucasian, and future research should address reporting accuracy and depression among different ethnic/racial groups.

Most importantly, our study identifies reporting accuracy as a potentially important factor in research concerning diet and depression. Higher depressive symptoms not only predict greater reporting accuracy in the general population, but our study demonstrates that depressive symptom severity also predicts greater reporting accuracy among individuals with major depressive disorder. Associations between depression and diet quality may be, in part, fueled by more accurate reporting of dietary intake on the part of individuals with depression. In order to inform treatment decision making when it comes to diet change among individuals with depression, future research should include an index of reporting accuracy in order to control for the influence of this factor on the appearance of dietary differences between euthymic and depressed individuals, and among those with relatively high, versus low, levels of depressive symptoms.
